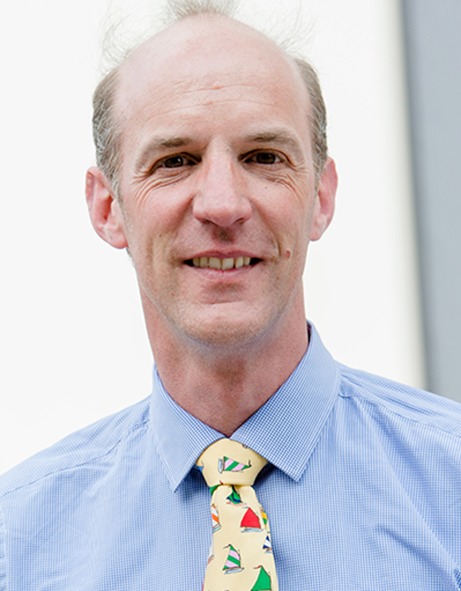# Letter from the editor

**DOI:** 10.1007/s00775-020-01756-5

**Published:** 2020-02-10

**Authors:** Nils Metzler-Nolte

**Affiliations:** Chair of Inorganic Chemistry I, Bioinorganic Chemistry, Faculty of Chemistry and Biochemistry, Ruhr University Bochum, Bochum, Germany

Dear JBIC Reader, dear Colleague:

I am excited to see the galley proofs of the first 2020 issue of JBIC, my first issue as Chief Editor of this journal. I am grateful to our previous Chief Editor, Larry Que, and Debbie Schoenholz as the editorial assistant, who were a superb team to lead and develop the journal for the past 20 years. Both have been professional and extremely helpful to enable a smooth transition with the beginning of this new decade.

Along with the change in the Editor’s office, you can expect more changes to come: On the Associate Editor’s team, Valeria Culotta has taken on a more demanding role in her institution with the beginning of 2020, and will no longer be handling manuscripts for JBIC. Thank you, Val, for your excellent service, and good luck with your new position. Efforts are underway to appoint a replacement for Val, and as with all of the latest news from JBIC, you will hear this first on our newly established Twitter account (@JBIC_Journal) – so follow the tweets!

It is my aim to maintain JBIC’s standing as the premier journal for new findings and developments in Bioinorganic Chemistry. JBIC serves as the major link within our global community, and it is our window to the outside world. Together with the Team of Editors we will work hard to increase its impact, and make sure that JBIC is a journal that all members of our society are proud of, and proud to publish in.

JBIC will maintain its roots in classical Bioinorganic and Medicinal Inorganic Chemistry, but we will also openly embrace new developments, for example in bioinorganic nanotechnology, the use of big data, or applications of digital chemistry to problems related to Bioinorganic chemistry. I would very much welcome suggestions for topics and authors, be it for reviews, commentaries and original articles, and of course I am always available for informal consultation before submission.

But finally, it is excellent science, hard, reliable data, and good service to our authors, reviewers and the community that will determine JBIC’s future standing. I am thus asking you to submit your excellent work to JBIC, cite articles in JBIC where appropriate, and make colleagues in neighbouring disciplines aware of us. At JBIC, we will make every effort to make your publishing experience with us a pleasant and rewarding one—so that it is with the same pleasure like mine today that you flip through the pages of every new issue of our journal.

Enjoy reading!

Nils Metzler-Nolte.

January 2020.